# The Immune Modulating Properties of Mucosal-Associated Invariant T Cells

**DOI:** 10.3389/fimmu.2020.01556

**Published:** 2020-08-13

**Authors:** Melina Ioannidis, Vincenzo Cerundolo, Mariolina Salio

**Affiliations:** Medical Research Council Human Immunology Unit, Medical Research Council Weatherall Institute of Molecular Medicine, University of Oxford, Oxford, United Kingdom

**Keywords:** MAIT cells, TCR-dependent, TCR-independent, dendritic cell maturation, inflammation, tissue repair

## Abstract

Mucosal-associated invariant T (MAIT) cells are unconventional T lymphocytes that express a semi-invariant T cell receptor (TCR) recognizing microbial vitamin B metabolites presented by the highly conserved major histocompatibility complex (MHC) class I like molecule, MR1. The vitamin B metabolites are produced by several commensal and pathogenic bacteria and yeast, but not viruses. Nevertheless, viral infections can trigger MAIT cell activation in a TCR-independent manner, through the release of pro-inflammatory cytokines by antigen-presenting cells (APCs). MAIT cells belong to the innate like T family of cells with a memory phenotype, which allows them to rapidly release Interferon (IFN)-γ, tumor necrosis factor (TNF)-α, and in some circumstances Interleukin (IL)-17 and IL-10, exerting an immunomodulatory role on the ensuing immune response, akin to iNKT cells and γδ T cells. Recent studies implicate MAIT cells in a variety of inflammatory, autoimmune diseases, and in cancer. In addition, through the analysis of the transcriptome of MAIT cells activated in different experimental conditions, an important function in tissue repair and control of immune homeostasis has emerged, shared with other innate-like T cells. In this review, we discuss these recent findings, focussing on the understanding of the molecular mechanisms underpinning MAIT cell activation and effector function in health and disease, which ultimately will aid in clinically harnessing this unique, not donor-restricted cell subtype.

## Introduction

MAIT cells are unconventional T lymphocytes that were first described by Porcelli et al. as one of two cell populations enriched in the CD4^−^ CD8^−^ T cell fraction (the other being Vα24^+^ iNKT cells) (1). It is now established that canonical MAIT cells express a semi-invariant TCRα-chain (in humans mostly Vα7.2-Jα33/Jα20, in mice Vα19-Jα33) paired with a number of TCR β-chains, contributing to a limited TCR repertoire (2, 3). Because of the limited TCR repertoire, and their similarities with iNKT cells, it was initially proposed by Tilloy and colleagues that these cells could be restricted by a non-classical MHC like molecule presenting either an endogenous ligand or a ubiquitous pathogen (4). Subsequently, it was demonstrated that MAIT cells are restricted to the highly conserved MHC-class I-related protein 1 (MR1) (5, 6). MR1 is β2m-associated, nonpolymorphic, and conserved across various mammalian species, with 90% of sequence similarity between mice and humans (7). The high inter-species conservation of MR1 and TCRα chain results in cross-reactivity between human and non-human species such as bovine, mouse, and rat (8, 9). Interestingly, a murine autoreactive MAIT hybridoma was shown to strongly recognize cells expressing bovine or rat MR1, but not human MR1, and this was pinpointed to residue Q151, present in the human but not the murine sequence (9). In the same study, polyclonal human MAIT cells were activated by rat, murine, and bovine MR1, but were not autoreactive (9).

MAIT cells preferentially locate in mucosal-associated tissue such as gut, lamina propria, and lung, in both humans and mice (6). Recent research has demonstrated that MAIT cells are also present in liver and human blood, where they can represent up to 50 and 10% of circulating CD8^+^ T cells, respectively (10, 11). Through the combined use of MR1 tetramers and Jα33^−/−^ mice, other populations of MR1-restricted T cells have been described, which express TCRs distinct from the canonical Vα7.2-Jα33, and may play antimicrobial as well as immunoregulatory functions (3, 11–13).

For many years after the discovery of MAIT cells restriction to MR1, the nature of the antigen MAIT cells detect in association with MR1 was unclear. In 2012, Kjer-Nielsen et al. demonstrated that MAIT cells TCRs recognize intermediates of the vitamin B2 (riboflavin) biosynthetic pathway (14). Several bacteria and fungi previously associated with MAIT cell activation (15, 16) produce agonist vitamin B2 metabolites that stimulate MAIT cell activation in a TCR-dependent manner. Viruses are unable to synthesize vitamin B2 metabolites and cannot elicit TCR-dependent MAIT cell activation. Nevertheless, viral infections can elicit MAIT cell activation in a TCR-independent manner, through the release of different cytokines such as IL-12 and IL-18 (17, 18). MAIT cell activation results in the production of a variety of chemokines and pro-inflammatory cytokines, associated with both Th1 (IFN-γ and TNF-α) (16, 19) and Th17 immunity (IL-17 and IL-22) (20), but in certain tissues or upon prolonged stimulation MAIT cells can also release IL-10 and IL-13 (21, 22). Like conventional T cells, cytokine secretion is controlled by key transcription factors, mainly T-bet and RORγt (23). In addition, MAIT cells efficiently lyse bacterially-infected epithelial cells through granzyme and perforin molecules (24, 25) and MAIT-derived granulysin and granzyme B may be effective against antibiotic resistant bacterial species (26).

Like iNKT cells, MAIT have a unique developmental pathway and their effector-memory phenotype is controlled by the master transcription factor PLZF (27). Several recent reviews have extensively discussed MAIT cell development, their antimicrobial role, and their contribution to cancer and inflammatory diseases (23, 28–30). Herein, we will discuss their role at the interface between innate and adaptive immunity and recent results describing an important contribution of MAIT cells to tissue homeostasis, with a view to potentially harnessing their immunomodulatory properties.

## Tight Regulation of MAIT Cell Activation

Like other populations of lymphocytes straddling across innate and adaptive immunity, such as iNKT and γδ T cells (31, 32), MAIT cells are emerging as important modulators of immune responses. As MAIT cells are particularly abundant at mucosal surfaces, where antigen might also be available at higher concentrations, tight regulation of their activity is required to avoid immunopathology: for example, accumulation of activated MAIT cells has been reported in the inflamed mucosa in ulcerative colitis (33) and in the gastric mucosa during *Helicobacter pilori* infection (34). Tight regulation of MAIT cell activity is likely to occur through several mechanisms, from regulation of MR1 expression, to antigen availability, stability, and modulation of MAIT cell activation through cognate interactions.

### MR1 Ligands and Their Importance in Modulating MAIT Cell Function

MR1 is ubiquitously expressed at the transcript level (7), although the protein is retained in the ER and surface expression is tightly regulated by antigen availability (35). The most potent natural MAIT cell agonists known to date are intermediates of the vitamin B2 biosynthetic pathway (14, 36), present in a number of bacteria, commensals and pathogenic (37, 38). Despite stabilizing MR1 molecules at the cell surface, folate derivatives are not recognized by the MAIT TCR (14, 36), although more in depth analysis with folate-loaded MR1 tetramers has identified small subsets of circulating TRAV1.2^+^ and TRAV1.2^−^ reactive T cells (3).

The structure-activity relationship of MR1 ligands has been well characterized and while MR1 surface upregulation correlates with the ability of the compounds to form a Schiff base with Lys43 of MR1, the agonist activity correlates with binding of the compound ribityl moiety to the TCR, via its Tyr95α residue (2, 39–41). Recently, a very elegant study with 20 altered metabolite ligands and 11 crystal structures of TCR-MR1-ligand ternary complexes has refined the molecular basis underpinning the potency and specificity of MAIT cell antigens, with the identification of an “interaction triad” between Tyr95α (in the MAIT TCR), Tyr152 (in the MR1 groove), and 5' and 2' OH groups in 5-OP-RU, which needs to be preserved for maximal agonist activity (42). These findings will be invaluable in future investigations exploring how to design ligands to better harness MAIT cell activity.

The full spectrum of MAIT cell ligands is still under appreciated, although two studies have reported agonist activity of drugs and drug like molecules (43) and of synthetic compounds identified *in silico* (44). The weak agonist activity of drugs like diclofenac and the antagonist activity of salicylates potentially underscores a much broader involvement of MAIT cells in several physio-pathological processes. Inhibitory ligands have the potential to be used to downregulate MAIT cell activation. Indeed, a synthetic derivative of the vitamin B9 metabolite 6-FP (i-6FP) has been used to inhibit MAIT cell activation and improve the course of the autoimmune disease lupus in FcγRIIb^−/−^ mice, a spontaneous model of systemic lupus erythematosus in which MAIT cells have been shown to enhance autoantibody production and tissue inflammation (45).

While the majority of antagonists stabilize MR1 through a Schiff base but lack a moiety capable of interacting with the MAIT TCR, a novel mechanism of inhibition has recently been identified (44). Two synthetic non-microbial compounds, DB28 and its derivative NV-18, retain MR1 in the endoplasmic reticulum in an immature ligand-receptive form and compete with stimulatory ligands for MR1 binding. Neither DB28 nor NV18 form a Schiff base with MR1, but they are both sequestered in the A' MR1 pocket by a network of hydrophobic and polar contacts (44).

### Antigen Stability

The potent MAIT cell antigens 5-(2-oxopropylideneamino)-6-D-ribitylaminouracil (5-OP-RU) and 5-(2-oxoethylideneamino)-6-D-ribitylaminouracil (5-OE-RU) derive from enzymatic and non-enzymatic condensation of 5-amino-6-(1-D-ribitylamino)uracil (5-A-RU) with glyoxals and methylglyoxals (host or bacteria derived). However, 5-OP-RU and 5-OE-RU are unstable and unless bound to MR1 via a Schiff base with Lys43 of the antigen presenting groove, they rapidly cyclize to less potent lumazines (36, 46). Furthermore, the biological activity of 5-A-RU is affected by long-term storage and spontaneous oxidation, unless prepared in dimethylsulfoxide solutions (46). To overcome the intrinsic instability of 5-A-RU, Lange et al. synthesized a pro-drug modifying the 5′ aminogroup with a cleavable valine-citrulline-p-aminobenzyl carbamate (47). The prodrug is stable and is cleaved intracellularly by cathepsin B, leading to preferential loading in the recycling endosomes.

### Antigen Availability

Antigen availability influences MAIT cell population expansion throughout life. 5-OP-RU MR1-tetramer binding cells are few at birth, but they rapidly increase within the first year of life, accounting for the majority of Vα7.2^+^ CD161^++^ cells in the circulation (48). Germ free mice lack MAIT cells (6, 49–51) and mono-colonization with riboflavin producing bacteria, or exposure to synthetic 5-OP-RU, is sufficient to rescue MAIT cell development (51). These results, although surprising in view of the instability of 5-OP-RU, underscore the high sensitivity of TCRs in detecting cognate antigens bound to the relevant antigen presenting molecule.

At steady state, microbial diversity and density increases from the upper to the lower gastrointestinal tract (52) and mucosal conditions affect the relative abundance of MAIT-stimulatory metabolites, thus influencing MAIT cell activation (37). It was shown that *E. coli* bacteria grown in anaerobic conditions, stationary phase, and in medium supplemented with glucose, xylose, ribose, or glycerol stimulated more potently MAIT cell activation (37). These growth conditions correlated with increased accumulation of stimulatory MAIT cell ligands, detected by mass spectrometry (37). Furthermore, location of bacteria in the luminal space vs. areas adjacent to the epithelium (such as for *bacteroides* spp., *proteobacteria*) is also likely to influence antigen availability (37). Finally, the relative expression of individual enzymes in the vitamin B2 biosynthetic pathway influences the balance of MAIT cell-activating and inhibitory metabolites, as shown for *Salmonella typhimurium* and *Streptococcus pneumoniae* isolates (53–55).

### Direct and Indirect MAIT Cell Activation

**Figure 1:**
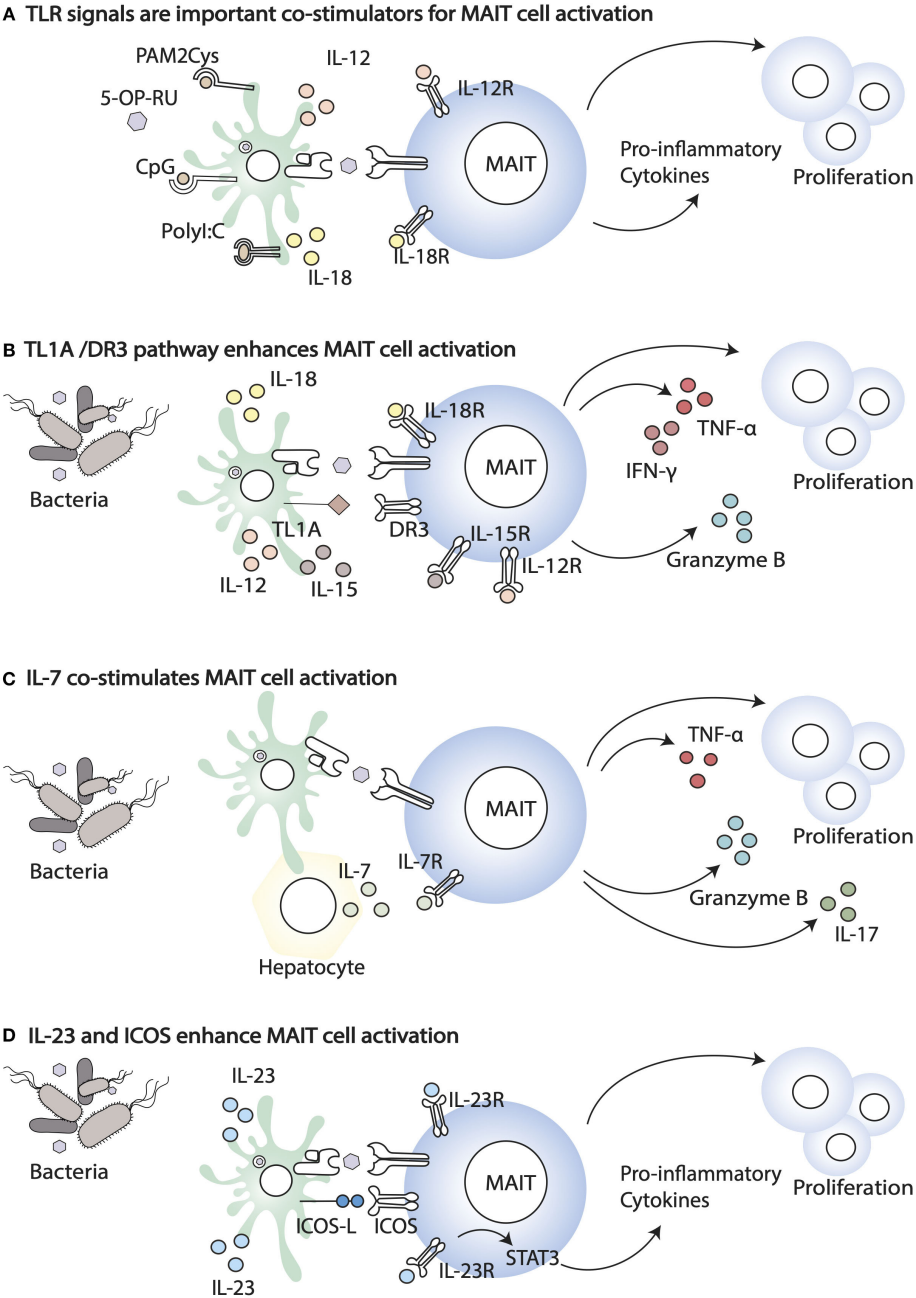
Co-stimulatory signals that enhance MAIT cell activation. **(A)** TLR agonists, including PAM2Cys, CpG, and PolyI:C, enhance MAIT cell response in the presence of the ligand. **(B)** Co-stimulation through the TL1A-DR3 pathway results in enhanced MAIT cell proliferation and the release of Granzyme B, IFN- γ, and TNF- α. **(C)** Infections trigger IL-7 release by different cells, including hepatocytes. IL-7 co-stimulates MAIT cells enabling cell proliferation and the release of IL-17, Granzyme B, and TNF- α. **(D)** Bacterial infection induces the expression of ICOS ligand (ICOSL) and ICOS (ICOS) on APC and MAIT, respectively. The increased expression of ICOS and IL23R signalling in MAIT cells enhance secretion of pro-inflammatory cytokines.

When the phenotype of MAIT cells from paired mucosal and blood samples has been analyzed, important differences have been highlighted. Colon resident MAIT cells are more activated (higher expression of CD137, CD69, HLA-DR, and CD25), but they also express higher levels of inhibitory receptors, such as TIGIT, CTLA-4, PD1, and LAG3 (37). This phenotype might reflect continuous exposure to metabolites derived from commensals and/or pathogenic bacteria and the expression of inhibitory receptors may balance this exposure. During bacterial infections, in addition to MR1-antigen complexes, MAIT cells are exposed to a variety of inflammatory cytokines that co-stimulate and enhance their activation, potentially overcoming inhibitory signals (Figure 1). The relative importance of TCR-driven vs. cytokine driven MAIT cell stimulation also changes during the course of an infection, with the former dominating at earlier stages of the response (56). Unlike conventional memory T cells, *in vitro*, MAIT cells are poorly responsive to anti CD3/anti CD28 stimulation, but their responsiveness is greatly enhanced by IL-12 and IL-18 (Figure 1A) (57). Freshly isolated blood MAIT cells and MAIT cell lines are potently activated by synthetic 5-OP-RU presented by myeloid cells, and in this setting their activation is mostly MR1-dependent (58). Yet, a high fraction of cells undergoes activation induced cell death, and perhaps cytokine dependent signals, including IL-12, IL-18, and IL-15, increase MAIT cell viability through changes in expression of pro and anti-apoptotic proteins, such as Bcl2 and Bax1 (59, 60).

The high expression of IL-12 and IL-18 receptors by MAIT cells [as well as by iNKT cells (61, 62)] facilitates their activation in a TCR-independent manner, during viral infections [reviewed in (63)]. In addition, cytokine stimulation is important during infections with *Group A streptococcus* bacteria, lacking the vitamin B2 biosynthetic pathway (64). In this paper the authors showed that MAIT cell activation occurs in response to streptococcal exotoxins of the superantigen family, recognized by the Vβ2 TCR chain, but independently of MR1 (64). Similarly, MAIT cell responsiveness to *Staphylococcus enterotoxin B* (SEB) superantigen occurs in a TCR Vβ13.2 dependent manner but is MR1-independent and is largely contributed by IL-12 and IL-18 (65).

The importance of cytokines signaling for achieving full MAIT effector function is also underscored by the observation that chronic stimulation by type I IFN (such as in HIV infected patients) results in impaired MAIT cell responses to bacteria, via IL-10-dependent suppression of IL-12 secretion by APCs (66). However, in other settings, type IFNs synergize with other signals to enhance MAIT cell activation, consistent with the notion that type I IFNs are an important third signal that shapes the differentiation of memory conventional T cells (67). Accordingly, in HCV infected patients treated with type I IFN and antivirals it was reported that MAIT cells express higher CD69, indicative of activation (17), and a synergistic activity of type I IFN and IL-15 was also demonstrated. Furthermore, type I IFN has also been shown to co-stimulate TCR dependent MAIT cell activation (68) in addition to the TCR independent activation discussed above (17).

Other inflammatory cytokines that have been shown to enhance MAIT cell responsiveness to antigen include the gut-associated TNF superfamily member TL1A (via death receptor 3, DR3 (Figure 1B) (69, 70) and IL-7 (Figure 1C). IL-7 is essential for proliferation of liver-derived MAIT cells, which like the colonic MAIT have a semi-activated state yet they have higher expression of negative regulators (SOCS1 and SOCS3), in addition to lower expression of TCR signaling components, which may be an adaptation to constant antigen exposure (71). Furthermore, in HIV patients, IL-7 has been shown to rescue the defective MAIT cell cytolytic capacity and cytokine secretion, both *in vitro* and *in vivo*, in patients on anti-retroviral therapy (72, 73).

*In vivo*, population expansion of MAIT cells following infection with *S. typhimurium* depends on riboflavin metabolites and microbial signals (74). Indeed, synthetic 5-OP-RU injection intranasally or intravenously is not sufficient to increase MAIT cell frequency and numbers in the lung and draining lymph nodes, unless it is accompanied by TLR-derived signals (Pam2Cys, polyI:C, or CpG) (Figure 1D). Likewise, infection with riboflavin-deficient *S. typhimurium* mutants does not lead to MAIT cells population expansion, unless complemented with synthetic 5-OP-RU (74). However, more recently, intraperitoneal injection (51) or skin application of 5-OP-RU (50) was shown to be sufficient to activate and expand MAIT cells, locally and systemically. Whether these discrepancies are due to differences in the microbial flora of the animal colonies of the different investigators, to different sensitivity of lung, skin vs. thymic MAIT cells, or to different antigen preparations and doses of antigens reaching the sites, it remains to be determined.

Nevertheless, for sustained expansion and cytokine secretion, some form of co-stimulation of MAIT cells seems to be required. Following cutaneous application of *S. epidermidis*, population expansion of MAIT cells is reduced in the absence of IL-18, but not IL-23, while IL-1 signaling is required for licensing of IL-17A production by MAIT cells (50). Using a mouse model of conditional MR1 targeting, the authors also demonstrated that MAIT cell population expansion following *S. epidermidis* application is MR1 dependent, although homeostatic maintenance of MAIT cells is not, as their frequency is unchanged 3 weeks post deletion of MR1.

Specific costimulatory requirements have been identified for population expansion of RORγt^+^ MAIT cells in response to intranasal *Salmonella typhimurium* or *Legionella longbeachae* (75). In this paper, through mixed bone marrow chimeras the authors showed that APCs-bone marrow derived and epithelial-are important in MR1-dependent antigen presentation. In addition, they demonstrated a role for ICOS-mediated co-stimulation and IL-23/IL-23R signaling in MAIT cell expansion and activation (Figure 1D). Both ICOS-deficiency and IL-23 deficiency impaired expansion mainly of the RORγt MAIT subset, suggesting they are required for maintenance of RORγt expression and IL-17 secretion. IL-23 was also shown to be sufficient on its own to co-stimulate MAIT cell proliferation, in the presence of 5-OP-RU and to up-regulate ICOS expression, although IL-23 and ICOS double deficient mice were not tested, nor was IL-23 measured in ICOS deficient mice (75). These results are consistent with the high expression of both ICOS and IL-23R in MAIT cells, compared to conventional T cells in naïve mice. In this context IL-23 signaling is important, presumably via STAT3, to maintain the Th17 signature of tissue resident MAIT cells. As homeostatic IL-23 contributes to MAIT cell development/accumulation in the presence of a normal microbial flora (50), these results also are consistent with the observation that patients with STAT3 loss of function mutations have, amongst others, a deficiency of MAIT cells (76).

## A TCR-dependent Tissue Repair Function of MAIT Cells

Overall, the above results are consistent with the hypothesis that due to ubiquitous MR1 expression and abundance of MAIT cells in tissues, their function needs to be tightly regulated, and for maximal effector function (i.e., during an infection) a combination of TCR, cytokine, and co-stimulation signals is required. In the absence of inflammatory signals, only minimal cell activation might be elicited. To understand the extent of MAIT cell functional plasticity and changes in their functional program in the course of an infection, several groups have analyzed their transcriptional signatures, in bulk and at the single cell level. At steady state, in both humans and mice, distinct transcriptional patterns have been identified, according to tissue specificity: despite different frequencies in different organs, MAIT/iNKT1 subsets, and MAIT/iNKT17 subsets share transcriptional signatures that are developmentally imprinted, including their tissue residency (77). Transcriptional changes occur at each developmental stage in the thymus and are underpinned by expression of the key transcription factors PLZF, T-bet and RORγt (27, 78), imprinting specific tissue residence signatures and Th1 or Th17 bias.

Recently, three groups compared the transcriptional signature of human and murine MAIT cells activated by bacterial infection (*Legionella longbeachae*), cytokines, and/or TCR stimulation (60, 69, 79). Despite differences in experimental models, common signatures were observed by the three groups. As expected from previous analysis at the protein level, upon activation, both human and murine MAIT cells expressed genes encoding for proinflammatory cytokines (such as GM-CSF, IL-17, INF-γ) and chemokines (such as XCL1, CCL3, CCL4, CXCL16) (60, 69). Human MAIT activated by anti-CD3/CD28 displayed a signature intermediate between unstimulated MAIT cells and those activated by cytokines and anti-CD3/28 (69). In turn, the transcriptional signature of human MAIT activated by cytokines and anti-CD3/CD28 (69) resembled that of 5-OP-RU activated human MAIT cells (60). Some species-specific differences were observed, such as higher expression of LIGHT and IL-2 in human MAIT cells, and higher TRANCE and RANKL in murine cells, but these could also be explained by different activation modes (60). Furthermore, and in line with their innate-like function, it was reported that the transcriptome of TCR-activated murine MAIT cells resembled that of iNKT cells, while upon resolution of infection it was more similar to γδ T cells (60). Interestingly, γδ T cells are a population of unconventional T cells that crucially contribute to tissue integrity and repair (80). Accordingly, an interesting finding common across these studies was the identification of a tissue repair signature upon TCR-dependent activation of MAIT cells. This signature was previously identified in H2M3 restricted CD8 Tc17 cells upon exposure to the commensal *S. epidermidis* (81) and was also seen in skin MAIT cells upon *S. epidermidis* topical application (50). Key genes in this signature are involved in tissue repair and remodeling (*MMP25, FURIN, PDGFB, TGFB1*) and angiogenesis (*CSF2, VEGFB, PDGFB*). Other MAIT cell-derived factors with potential role of tissue homeostasis were IL-26, OSM, and HBEGF (69, 79). Hence an important function of tissue resident MAIT cells could be maintenance of tissue homeostasis in the presence of commensals, limiting inflammation, and associated tissue injury. In agreement with these findings, MAIT cells in the female genital tract, in the oral mucosa, and in fetal mucosal tissues show a bias toward IL-22 and IL-17 secretion, and a potential role in barrier immunity and tissue homeostasis (20, 82, 83). This hypothesis is also consistent with *in vitro* results demonstrating that supernatants of activated MAIT cells promote closure of scratches of monolayers of Caco2 cells (69) and *in vivo* results showing that MR1 deficient mice have reduced re-epithelization of skin wounds (50). In addition, the MAIT tissue repair function could account for increased gut permeability in MR1^−/−^ NOD mice, compared with MR1 sufficient littermates (84). Finally, alteration of gut permeability and MAIT cell numbers following conditioning regimens and allogeneic bone marrow transplantation could contribute to intestinal symptoms of Graft versus host disease (GvHD) (85, 86). In the future it will be interesting to investigate whether there is an alteration in the homeostatic tissue repair function of MAIT cells in chronic fibrotic diseases, in which a pathogenic MAIT cell role has been suggested (87–89).

## MAIT Cells: The New iNKT/γδ T Cells?

**Figure 2:**
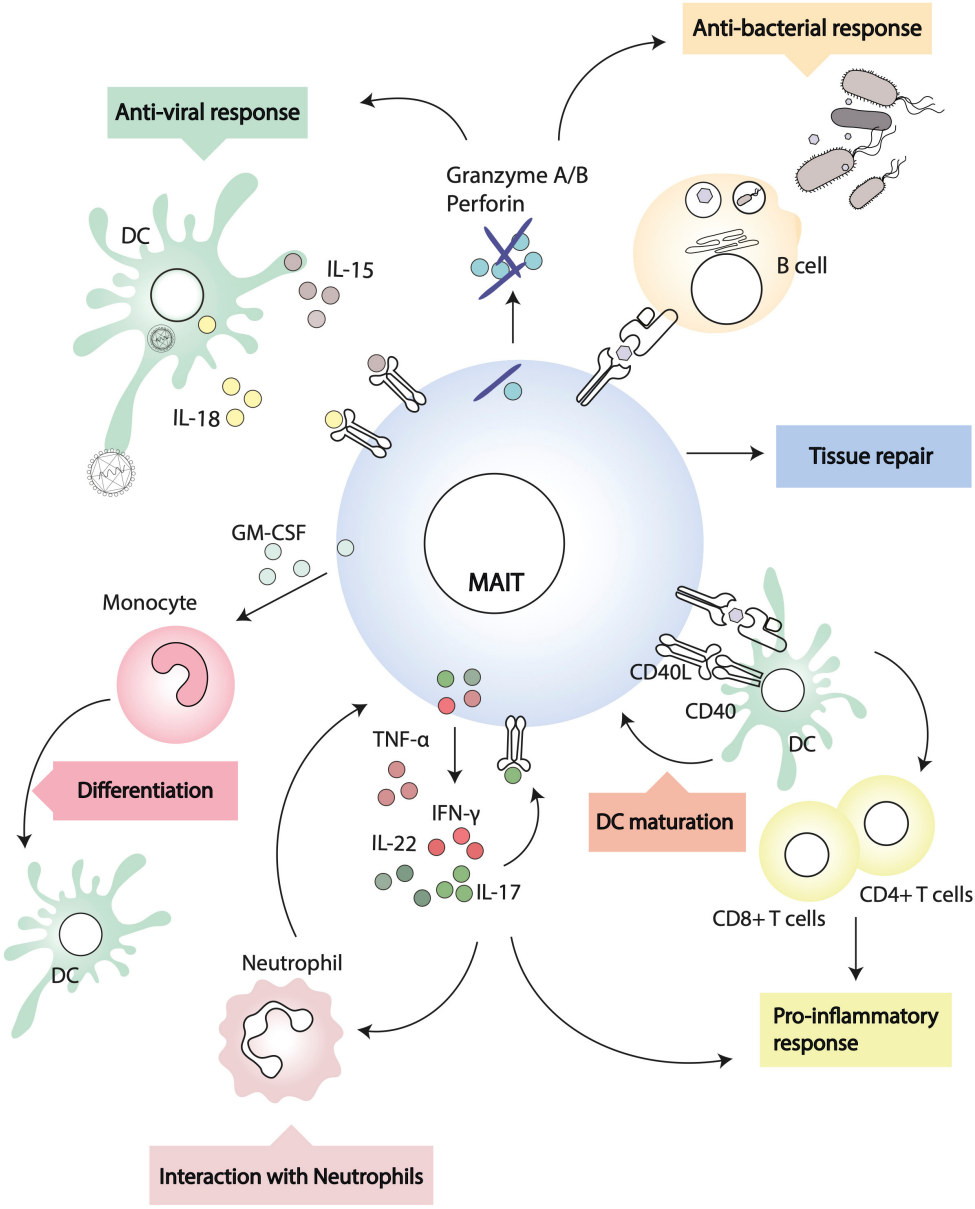
Interactions of MAIT cells and other leukocytes. MAIT cells recognize MR1-antigen complexes on the surface of target cells and modulate their activity through cognate interactions (i.e., via CD40/CD40L) or through soluble factors.

Common features across the three major populations of innate-like lymphocytes—iNKT, γδ, and MAIT cells—are their peculiar thymic differentiation program, after which the cells emerge as pre-set memory/effectors, poised to a rapid response upon antigen encounter (31, 32, 90, 91). Through secretion of a variety of cytokines and chemokines, iNKT cells and γδ T cells regulate the function of several immune cell subsets and have emerged as central players in immunobiology and immunopathology, bridging innate and adaptive responses (Figure 2). We will discuss the existing evidence in favor of the immunomodulatory activity of MAIT cells.

### Interactions With Myeloid Cells

MAIT cells can modulate myeloid cell function directly, following MR1-cognate interactions, or indirectly, through soluble factors. During *in vivo* pulmonary infection with *Francisella tularensis* live vaccine stain (LVS), it has been shown that MAIT cells produce critical antimicrobial cytokines (IFN-γ, IL-17A, TNF-α) and in MR1^−/−^ mice there is a higher bacterial burden and delayed bacterial clearance (92). In addition, through early GM-CSF production, pulmonary MAIT cells promote the differentiation of CCR2^+^ inflammatory monocytes into dendritic cells (DC) (93). As DC are critical for priming adaptive immunity, recruitment of activated TCRβ^+^ CD4^+^ and CD8^+^ cells is significantly delayed in MR1 deficient mice (92), but it can be rescued by adoptive transfer of *in vivo* differentiated DC (93). In this experimental system, MAIT cells influence monocyte differentiation into DC in a GM-CSF dependent but MR1 independent way (93). In other models, however, MAIT cells can modulate DC function in an MR1-dependent manner. Indeed, it has been shown that primary human MAIT cells and MAIT cell lines induce maturation and activation of monocyte-derived DC and primary DC upon MR1-dependent recognition of 5-OP-RU complexes (58). DC maturation is dependent on CD40-CD40L signaling and results in secretion of bioactive IL-12, which can further modulate NK cell activation (58). Interestingly, murine MAIT cells can also upregulate CD40L upon activation (75), although it remains to be determined whether they are able to induce antigen specific adaptive T cell responses through CD40L dependent DC maturation, as is the case for iNKT cells (94, 95).

Additionally, MAIT cells have been shown to exert a protective function in non-alcoholic fatty liver disease, inducing M2-macrophages polarization through IL-4 secretion (96).

### Interactions With B Cells

MAIT cells final differentiation and peripheral expansion depends on cognate interactions with B cells in mice, although this does not seem to be critical in humans (6, 97). MAIT can be directly activated by bacterial infected B cells with up-regulation of CD69, and secretion of IFN-γ, TNF-α, and IL-17 (98). The observation that in *Vibrio cholera* infection (99) and *Shigella dysenteriae* vaccination (24) the circulating frequency of MAIT cells correlates with the pathogen-specific antibody response suggests some form of helper activity from MAIT cells. In a murine model of lupus, MAIT cell activity correlates with autoantibodies, germinal center reaction, and severity of disease (45). In this paper, the authors also reported that, *in vitro*, MAIT cells enhanced IgG production by LPS-stimulated B cells through CD40-CD40L cognate interactions (45).

*In vitro*, MAIT cell supernatants have been shown to induce plasmablast differentiation and antibody secretion from memory B cells (100). In this system, help for B cells is provided in an MR1-dependent, but CD40L independent manner, likely via cytokines like IL-6, IL-10, and IL-21 (100). However, the authors only blocked soluble CD40L and did not address whether during cognate interaction between B cells and MAIT cells the CD40/CD40L axis has a role for B cell differentiation. Furthermore, as the CD161 ligand LLT1 has been shown to play a role in the germinal center reaction (101), there remains the possibility that CD161^++^ MAIT cells, in addition to the more abundant CD161^+^ follicular DC subset, might contribute to B cell differentiation through cognate interactions. Lastly, it is not yet established whether a T-follicular helper subset of MAIT cells exists, akin to Bcl-6^+^ iNKT cells providing cognate B cell help in the lymph node (102–104).

### Interactions With Neutrophils

In the presence of monocytes, MAIT (and γδ) T cells rapidly respond to bacterial infected neutrophils, and through secretion of GM-CSF, IFN-γ, and TNF-α they increase neutrophil survival and promote their differentiation into antigen presenting cells, expressing CD64, CD83, HLADR, CD54, CD40, and HLA A, B, C (105). In addition, in this manuscript, MAIT and γδ T cells activated neutrophils acquired the ability to uptake exogenous antigens, and cross-present antigenic peptides to CD8^+^ T cell clones (105). The ability of MAIT cells to modulate neutrophils function could play an important role in sepsis patients (106), where the number of circulating MAIT cells might affect the outcome of infection, particularly in the aging population with declining numbers of MAIT cells (107). Furthermore, as MAIT cells can become anergic upon recognition of microbial superantigens that often are associated with sepsis (65), and are then impaired in their ability to respond to bacterial infected cells, licensing of activated neutrophils to present antigens to CD4^+^ and CD8^+^ T cells may become crucial for the establishment of protective immunity (105).

Contradicting the above results, a recent study found that neutrophils inhibit MAIT cell activation through cell contact and hydrogen peroxide and that MAIT cells-derived TNF-α induces neutrophil death (108). The discrepancies might be related to different experimental settings, for example, one study isolated neutrophils by dextran sedimentation followed by Ficoll-Plaque centrifugation and hypotonic lysis of remaining red blood cells, while the other study purified them from whole blood or Lymphoprep separated granulocytes by HetaSep sedimentation and negative selection with the EasySep neutrophil enrichment kit; one study activated MAIT cells with 5-OP-RU, while the other with anti-CD3/28 beads, hence the amount of cytokines in the supernatants would be different. Therefore, further research is required to understand the exact outcome of MAIT-neutrophils interactions.

It also remains to be determined whether, like iNKT cells, MAIT cells are able to modulate the suppressive function of granulocytic myeloid derived suppressor cells, which could be of relevance to relive cancer immunosuppression and amenable to clinical harnessing (109, 110).

## Concluding Remarks

Because of their high numbers in humans and of the restriction by a monomorphic molecule, MAIT cells represent an interesting population to target to enhance antigen specific immunity, through their multiple interactions with cells of the innate immune system. Despite great progress since the first discovery of T cell populations bearing semi-invariant TCRs, several aspects of MAIT cell biology remain to be fully unraveled before harnessing MAIT cells can be taken forward into the clinic. We need to better understand the role of MAIT cells in several diseases in which their numbers or functionality is altered, such as cancers and autoimmune diseases. The molecular mechanisms of ligand antigen presentation are not completely defined and a characterization of endogenous antigens, if they exist, is still lacking. Known ligands occupy the MR1 A' pocket and it remains to be determined if ligands or chaperones will bind the F' pocket. Pathogen evasion mechanisms from MAIT immune-surveillance have been identified (55, 111), but further research in this field is also needed. Lastly, the recently described MAIT tissue-repair function needs to be molecularly defined as well as the interplay with the host microbiota, which is key in homeostasis.

## Author Contributions

MI wrote the first draft. MS edited and extended it. The paper is dedicated to the memory of VC. All authors contributed to the article and approved the submitted version.

## Conflict of Interest

The authors declare that the research was conducted in the absence of any commercial or financial relationships that could be construed as a potential conflict of interest. The handling Editor declared a past co-authorship with one of the authors VC.
